# Epidemiological modeling of *Trypanosoma cruzi*: Low stercorarian transmission and failure of host adaptive immunity explain the frequency of mixed infections in humans

**DOI:** 10.1371/journal.pcbi.1005532

**Published:** 2017-05-08

**Authors:** Nicolás Tomasini, Paula Gabriela Ragone, Sébastien Gourbière, Juan Pablo Aparicio, Patricio Diosque

**Affiliations:** 1 Instituto de Patología Experimental, Facultad de Ciencias de la Salud, CONICET, Universidad Nacional de Salta, Salta, Argentina; 2 UMR 228 ESPACE-DEV-IMAGES, ‘Institut de Modélisation et d'Analyses en Géo-Environnement et Santé’, Université de Perpignan Via Domitia, Perpignan, France; 3 Instituto de Investigaciones en Energía no Convencional, CONICET, Universidad Nacional de Salta, Salta, Argentina; ETH Zurich, SWITZERLAND

## Abstract

People living in areas with active vector-borne transmission of Chagas disease have multiple contacts with its causative agent, *Trypanosoma cruzi*. Reinfections by *T*. *cruzi* are possible at least in animal models leading to lower or even hardly detectable parasitaemia. In humans, although reinfections are thought to have major public health implications by increasing the risk of chronic manifestations of the disease, there is little quantitative knowledge about their frequency and the timing of parasite re-inoculation in the course of the disease. Here, we implemented stochastic agent-based models i) to estimate the rate of re-inoculation in humans and ii) to assess how frequent are reinfections during the acute and chronic stages of the disease according to alternative hypotheses on the adaptive immune response following a primary infection. By using a hybrid genetic algorithm, the models were fitted to epidemiological data of Argentinean rural villages where mixed infections by different genotypes of *T*. *cruzi* reach 56% in humans. To explain this percentage, the best model predicted 0.032 (0.008–0.042) annual reinfections per individual with 98.4% of them occurring in the chronic phase. In addition, the parasite escapes to the adaptive immune response mounted after the primary infection in at least 20% of the events of re-inoculation. With these low annual rates, the risks of reinfection during the typically long chronic stage of the disease stand around 14% (4%-18%) and 60% (21%-70%) after 5 and 30 years, with most individuals being re-infected 1–3 times overall. These low rates are better explained by the weak efficiency of the stercorarian mode of transmission than a highly efficient adaptive immune response. Those estimates are of particular interest for vaccine development and for our understanding of the higher risk of chronic disease manifestations suffered by infected people living in endemic areas.

## Author summary

Chagas disease is caused by a unicellular parasite mainly transmitted by blood-feeding bugs. The disease starts with an acute phase when the parasite multiplies in tissues. The immune system is then activated and almost clears the parasites before the host enters the chronic phase of the disease. As hosts remain exposed to the vectors, secondary contacts may result in re-inoculation with the parasite. The re-inoculated parasites then have to face an adaptive immune response that may prevent reinfection. There is little quantitative knowledge about the frequency of such re-inoculation and the efficiency of the immune system to prevent them. Since reinfections can lead to people carrying two or more strains of the parasite, we can potentially infer on the frequency of reinfection from the detection of such mixed infections. We fitted agent-based models to field data to estimate the frequency of reinfection in humans and its timing after primary infection. Our models predicted that most reinfections occur during the chronic phase as the adaptive immune response fails to prevent them in 20% of re-inoculations. Such a rate of failure is particularly important as reinfections are thought to increase the risk of heart damage in the chronic phase of the disease.

## Introduction

Chagas disease (American trypanosomiasis) is a vector-borne disease primarily transmitted by a broad range of ecologically and evolutionary diversified triatomine species (e.g. [1]). Despite multinational control initiatives [2], vectorial transmission is still active in several countries and the disease remains a major public health concern in Latin America, where several million people are at risk of infection.

The disease is characterized by a persistent infection with the parasite *Trypanosoma cruzi*. High parasitaemia is typically observed in the acute stage of infection until the immune system establishes an efficient, albeit not complete, control of the pathogen. Finally, the disease enters a chronic stage where parasite persistence is associated with chronic manifestations of the disease [3–8]. In addition, this parasite persistence triggers a constant activation of the immune system that is thought to exert a resistance to reinfections. This condition of non-sterile immunity, known as premunition or premunity [9], has been described for several diseases like malaria [10–12] and infections by helminthes [13–17]. Despite such a potential immune response, there are evidences that reinfections actually occur in animal models of Chagas disease such as mice [18–22], dogs [23], guinea pigs [24] and hamsters [25]. Evidences about reinfections in humans are more restricted. Macedo and coworkers reported a possible case of reinfection of a previously diagnosed man who developed an acute chagasic myocarditis in a longitudinal study [26]. Additional evidences about reinfections in humans are indirect and based on transversal studies assuming the vector exposure time as a synonymous of occurrence of reinfections [27–29]. Several authors proposed a role of reinfections in pathogeny. Although parasitaemia is lower in the reinfections compared to the primary infection in animal models [20, 22, 23], reinfections may aggravate chronic disease in mice [20–22, 30, 31] and they may be associated to chronic cardiac damage in humans [28, 29, 32]. To determine the frequency of reinfections and the ability of the human immune system to prevent them is thus essential to our understanding of the disease dynamics. In addition, it may help approaches for vaccine development. Since reinfections in humans cannot be experimentally evaluated for obvious ethical concerns, mathematical and computational models of Chagas disease transmission are critically needed to infer on the rate and timing of *T*. *cruzi* reinfections from epidemiological field data.

Reinfections can cause mixed infections, i.e. infection with at least two different genotypes of the same parasite. The analysis of mixed infections by *T*. *cruzi* may thus help gaining the desired knowledge about human reinfections, provided that one is able to establish quantitative predictions about the expected patterns of such mixed infections. We thus identified a complete set of four hypotheses that can explain the occurrence of a mixed infection in a human. Under a first hypothesis that we refer to as “Full Protection” (FP), no new parasite can be permanently established in the host after a primary infection. A mixed infection can then only result from the vector transmitting more than one lineage in the primary infective contact. Second, under an “Acute-Phase Window” (APW) hypothesis, the adaptive immune response is not completely established during the acute stage. Consequently, reinfections by different lineages can then cause a mixed infection in this stage. Alternatively, reinfections can be negligible in the short time of the acute phase, but they can occur in the chronic phase if the adaptive immune response fails. We called this third hypothesis “Protection Failure” (PF). The last hypothesis is a combination of the APW and the PF, so that reinfections may occur in both phases of the disease. Such reinfections can cause mixed-infections.

In this paper, these four alternative hypotheses are modeled to predict the transmission of *T*. *cruzi* major lineages (called ‘discrete typing units’—DTUs) [33, 34] that were detected circulating in Chaco province, Argentina [35–37]. The predicted rates of mixed-infections are compared to the observed pattern to infer on the rate of reinfection and to discriminate which of the four different hypotheses about the host immune response has major relevance. This provides a simple conceptual background based on agent-based modeling in order to infer on the rate of re-inoculations and the rate and timing at which re-inoculated parasites evade the immune system to reach persistence in humans.

## Methods

### General overview of the methods

To test the four different hypotheses (FP, APW, PF and APW + PF), we developed an agent-based model (ABM) of *T*. *cruzi* transmission. Briefly, an ABM is a computational model that includes two main components: the environment and the agents. Our models simulated *T*. *cruzi* transmission in an environment corresponding to a rural village made of contiguous houses. Houses had different agents representing humans and bugs. These agents may have different states (infected or non-infected). In addition, infected agents are infected by one or more DTUs. Different rules were specified to describe the population dynamics of the agents, according to typical birth, death and dispersal processes. In addition, other rules determined the transmission of the parasites between hosts and vectors. Four different models were set up by specifying transmission rules according to each of the hypotheses about the immune response and the origin of reinfection (FP, APW, PF and APW + PF). For any set of demographic and transmission parameters, the models were run until the prevalence of infection in humans (*P*_*H*_) and in vectors (*P*_*V*_), and the frequency of mixed infections in humans (*F*_*mix*_) had reached their equilibrium. Different approaches were then used to compare the predictions obtained from the models and the epidemiological situation observed in the populations studied in the Gran Chaco. First, we fitted the models to the observed *P*_*V*_, *P*_*H*_ and *F*_*mix*_ values. Predicted prevalences and likelihoods of the best fitted models were compared to evaluate the ability of each of the hypotheses to reproduce the observed patterns. Second, we calculated the probability for the different models to predict an *F*_*mix*_ as high as the observed value when values of different model parameters (i.e. mortality rates, transmission probabilities, etc.) freely vary within plausible biological ranges, which we refer to as uncertainty analysis. Third, a sensitivity analysis was made to determine the specific influence of each parameter on the value of *F*_*mix*_. Finally, because intra-domestic reservoirs can potentially affect the prevalence and frequency of mixed infections, we evaluated the robustness of our conclusions to the inclusion of such a reservoir host. This model fitting and comparison approach allowed assessing the ability of the four hypotheses to explain the infection patterns observed in the studied village of Chaco and to concomitantly provide the first quantitative estimates of the rate of reinfection in humans and the frequency of such reinfections in the acute and in the chronic stage of the disease.

### The Agent Based Model (ABM) of *T*. *cruzi* transmission

The components and the dynamical rules of our model are described below. Table 1 provides a summary of all parameters including their definition and their typical range of values as reported in the literature.

**Table 1:** Parameters of the Agent Based Models of transmission of *Trypanosoma cruzi*.

Parameter	Description	Range/value	Reference/justification
**Host and vector population parameters**			
***C***	Number of houses	100	Typical number of houses in rural villages from Chaco
***H***	Number of humans per house	5	[38]
***V***	Number of vectors per house	50–500	[39]
*B*	Feeding rate of vectors on mammals	0.09–0.31 feeding contacts /bug/day in spring and summer^1^.	[40]
***M*_*V*_**	Mortality of vectors	0.0045–0.0083 deaths /bug/day	Corresponding to a life expectancy of 120–220 days according to mortality of 4-5th instars and adults [41]
***M*_*H*_**	Mortality (plus emigration from village) of humans	6.8 x 10^−5^–13 x 10^−5^ events /human/day	Corresponding to an average lifetime in the village of 21–45 years.
***m***	Migration rate of vectors	0–0.02 events/vector/day	Upper value set as twice the observed value [42]
***N***	Nymph stage duration	60 days	Duration of 4-5th instars [41].
***T*. *cruzi* transmission parameters**			
***T*_*H->V*_**	Probability of transmission from human to vector per contact	0.005–0.06	[43]
***T*_*V->H*_**	Probability of transmission from vector to human per contact	2.6 x10^-4^–11 x10^-4^	[39]
***T*_*mix*,*H->V*_**	Probability of transmission of a mixed infection from human (with multiple DTUs) to vector	0.4–0.9	Upper value set as under experimental conditions [44] and unknown lower value set arbitrarily.
***T*_*mix*,*V->H*_**	Probability of transmission of a mixed infection from vector (with multiple DTUs) to human	0.4–0.9	The range was set equal to *T*_*mix*,*H->V*_
*A*	Acute phase duration	0–90 days	[45]
***F***	Protection failure rate in the chronic phase: probability of reinfection after re-inoculation	0–1	

^1^ the minimum feeding rate (on humans) was set as the mean of vector biting rates in spring and summer multiplied by the human blood index as determined in [40].

#### Environment and agents

The environment is modeled by a squared grid of cells where each cell represents a house. The dimension of the grid and the number of houses were set to C = 10 x 10. Because transmission of *T*. *cruzi* to humans is maximal in spring and summer [38], we accounted for seasonal transmission by allowing for new infections only during this six months period. The human and vector agents are characterized by state variables. Humans and vectors may be infected or not. Infected humans may be in the acute or the chronic phase of the disease, and vectors can be nymphs or adults. Finally, all infected agents contain one or more *T*. *cruzi* DTUs (TcI, TcIII, TcV and TcVI).

#### Population dynamics

The per house numbers of hosts (*H*) and vectors (*V*) were kept as constant, which implied replacing any died agent by a new uninfected agent. This typical assumption has been adopted for various models of *T*. *cruzi* transmission that include another agent-based model of *T*. *cruzi* transmission [46] and compartmental models (see [47] for a review). The modeling of population dynamics is then down to the description of mortality, development and migration processes. The host and vector mortalities (*M*_*V*_ *and M*_*H*_) were both considered as constant, i.e. they were affected neither by seasonality nor by any individual status (infection or stage of development), and vector development was modeled by assuming that nymphs become adults after *N* days. Humans did not disperse within the grid as we assumed that individuals sleep in the same house every night, where they can potentially be infected. Only adult vectors could disperse with an *m* probability. We mimicked seasonal dispersal by setting *m*>0 in spring and summer, and *m = 0* during the rest of the year. To keep the model simple, the vector population size was constant in each house. Consequently, we modeled dispersal as a switch of vector individuals between any two randomly selected houses (from the entire grid) and we used an *m*/2 rate because the switch implies migration of two individuals.

#### Transmission dynamics

**Fig 1:**
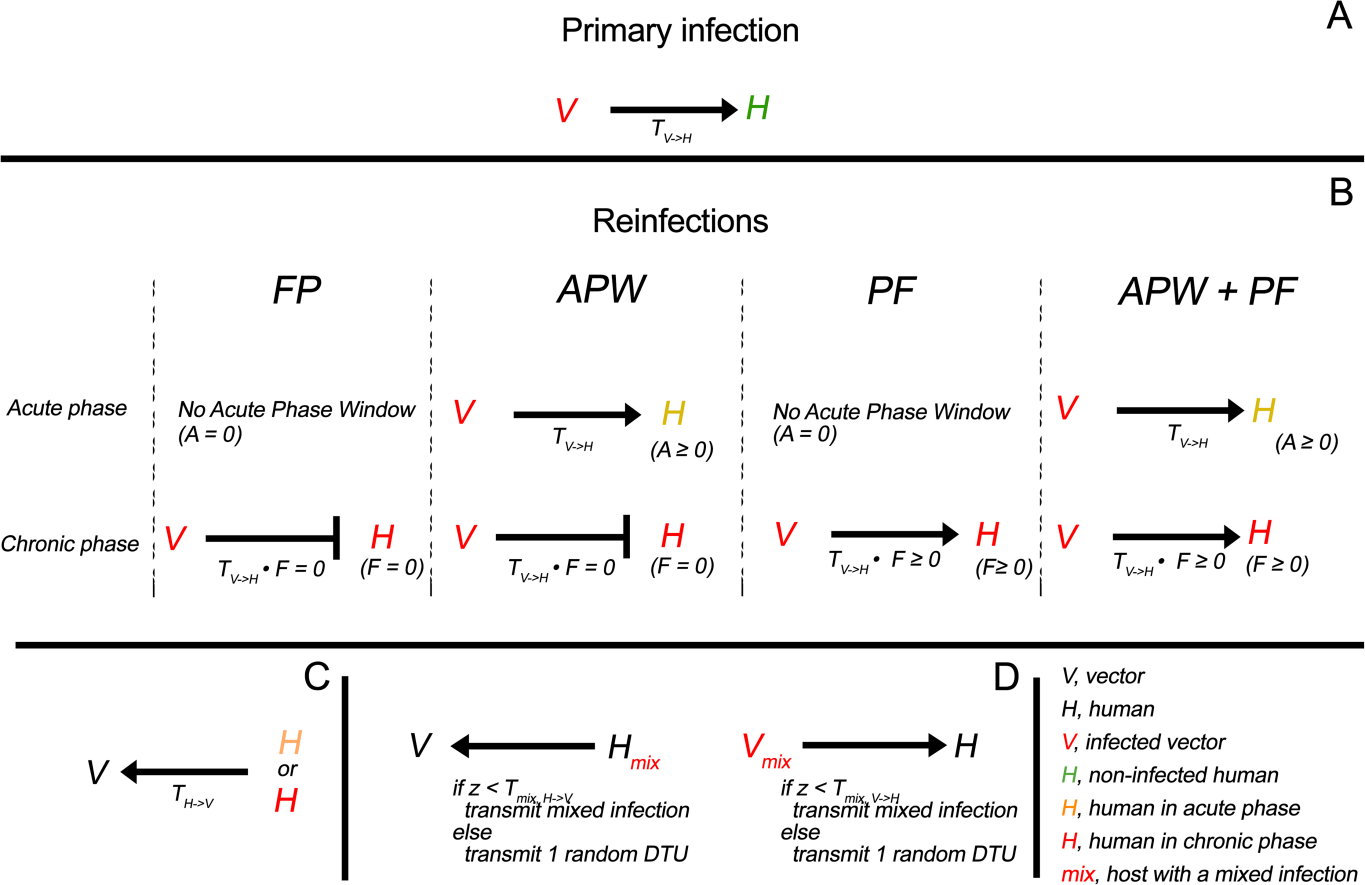
Rules of *T*. *cruzi* transmission according to the four hypotheses on reinfections. (A) Transmission from vector to uninfected humans with probability *T*_*V→H*_. (B) Definition of the four rules of reinfections. Full Protection *(FP*); Acute Phase Window (*APW*); Protection Failure *(PF*); Acute Phase Window and Protection Failure (*APW + PF*); *A*, acute phase window duration; *F*, protection failure rate. (C) Transmission from human to vectors with probability *T*_*H→V*_. (D) Transmission of mixed infections. *T*_*mix*_ is the probability of transmission of a mixed infection and *z* a random number uniformly distributed between 0 and 1. The transmission of the mixed infection occurs when z < *T*_*mix*_, and a single randomly selected DTU is transmitted otherwise.

The observed seasonality in transmission [38] was modeled by considering a rate of contact per day between vectors and mammals (*B)* varying between the two seasons, with *B*>0 in spring and summer and *B* = 0 in the rest of the year. The different transmission rules that described our four hypotheses about the origin of mixed infection are represented in Fig 1. In each potential infectious contact between an infected vector and a non-infected human, a primary infection may begin with *T*_*V→H*_ probability (Fig 1A). Infected individuals can then be potentially re-infected either in the acute or in the chronic phase or both phases of the disease (Fig 1B). During the acute phase window, whose duration is represented by *A*, the per-contact probability of reinfection is identical to the rate of primary infection *T*_*V→H*_, while in the chronic stage this probability is weighted by a factor *F*, where *F* is the rate of protection failure. Within this framework, the ‘FP’ hypothesis was modeled by setting *A* = 0 and *F* = 0, so that, in model ‘*FP’*, no reinfection was allowed either in the acute phase or in the chronic phase. The ‘APW’ hypothesis was described in model ‘*APW’*, by allowing *A* to take on positive values, while reinfections were still prevented in the chronic phase by setting *F* = 0. The ‘PF’ hypothesis and model ‘*PF’* was defined by setting *A* = 0 (no acute phase window), and allowing for reinfections in the chronic phase by letting *F* to be positive. The ‘APW + PF’ hypothesis was accounted for in model ‘*APW + PF’* as *A* and *F* can take on positive values. Parasite transmission to vectors occurred with a probability *T*_*H→V*_ (Fig 1C) that we assumed to be constant. When transmission occurred from an individual (host or vector) carrying a mixed-infection, it involved either a single or several DTUs. We denoted *T*_*mix*,*V→H*_ and *T*_*mix*,*H→V*_, the probability of transmission of the mixed infection from an infected vector and from an infected human, respectively (Fig 1D). When the transmission of a mixed infection had occurred according to *T*_*mix*_, all DTUs were transmitted (i.e. a human infected with TcI, TcIII and TcV that transmit all their DTUs to the vector), otherwise only one randomly picked DTU was transferred (i.e. just TcI, TcIII or TcV is transmitted with probability 1—*T*_*mix*_).

### Epidemiological field data

The different models were fitted to a previously published epidemiological dataset from two neighbor rural settlements (El Palmar and Las Leonas) located in the Chaco Province of Argentina, where vector-borne transmission is still active [37]. Prevalence data and frequencies of mixed infections measured between 2008 and 2010 are presented in Table 2, and were used as initial conditions to run the models. The prevalence in 276 vectors (*Triatoma infestans*) captured from houses was determined by detection of the parasite in the bug faeces under microscopic observation.

**Table 2:** Prevalence of infection in humans, dogs and vectors.

Prevalence of infection	% (95% CI)†
***Prevalence in human (P*_*H*_*)*^1^**	44.1 (39.9–48.3)
***Prevalence in vector (P*_*V*_*)*^2^**	17.0 (13.1–21.9)
***Prevalence in dogs (P*_*V*_*)*^3^**	20.4 (16.4–25.2)
***Prevalence of single infection in humans (F*_*single*_*)***	**43.4 (33.2–54.1)**
**TcI**	1.2 (0.2–6.5)
**TcIII**	0.0 (0.0–4.4)
**TcV**	36.1 (26.6–46.9)
**TcVI**	6 (2.6–13.3)
***Prevalence of mixed infection in humans (F*_*mix*_*)*^4^**	**56.6 (45.9–66.8)**
**TcI/TcV**	3.6 (1.2–10.1)
**TcI/TcVI**	1.2 (0.2–6.5)
**TcIII/TcV**	1.2 (0.2–6.5)
**TcV/TcVI**	38.6 (28.8–49.3)
**TcI/TcV/TcVI**	12.0 (6.7–20.8)

^1^ Calculated as the percentage of people with at least two positive serological tests.

^2^ Determined by microscopic observation of faeces collected from intra-domiciliary bugs.

^3^ Estimated by using ELISA-test.

^4^ Percentage of mixed infections among individuals with positive kDNA-PCR (infected individuals according to diagnostic PCR).

†Confidence intervals calculated from the Wilson binomial approximation for a proportion [48].

#### Model fitting by using a hybrid genetic algorithm

We implemented a hybrid genetic algorithm in order to fit each model to the observed prevalences: *P*_*H*_, *P*_*V*_ and *F*_*mix*_. A genetic algorithm (GA) is a global optimization method based on the idea of natural evolution in order to select the combination of parameter values that maximizes a fitness function. The potential solution of a problem is encoded on an ordered data structure called chromosome, and the algorithm works on a population of several chromosomes [49]. Here, the population size was set to 50 chromosomes and the combinations of parameters values bear by each of those chromosomes were randomly generated by using a Latin hypercube sampling. Each chromosome was evaluated for their fitness using simulated likelihood calculated as follow. Average predictions for *P*_*H*_, *P*_*V*_ and *F*_*mix*_ were obtained from 1,000 simulations of the ABM ran with the parameter values bear by the chromosome. The probability mass function of the binomial distribution was then used to estimate the likelihood of such predicted values conditioned on the observed values in the host and vector populations. For example, the likelihood of the predicted *P*_*H*_ is calculated by using probability mass function with the observed number of infected people (number of successes), the population size (number of trials) and *P*_*H*_ (probability of success) as parameters of the function. The product of such likelihoods was used as a measure of fitness. Different operators (i.e. selection, recombination and mutation) are applied to the population of chromosomes every generation in order to ‘evolve’ towards an optimal solution. First, the twenty-five chromosomes with best likelihood were selected to populate the next generation in the algorithm and the remaining were discarded. A ‘clonality plus mutation’ operator was then applied to the six best chromosomes to simulate a simple hill-climbing algorithm and speed up the search for the global optimum [50]. Briefly, each selected chromosome was copied and mutated in a single parameter at random to generate a descendant chromosome. The mutation consisted on the replacement of the selected parameter value by another random value. However, in order to get a cloned chromosome which solution is a neighbor of the solution of the parental chromosome (a step of a hill-climbing algorithm), the mutation was limited such that the change is as maximum 10% of the parameter value. The second operator was recombination. The twenty-five best chromosomes were selected to recombine using a roulette wheel approach with a single-point recombination [51] until the next generation of the chromosome population is complete [52]. Third, the mutation operator was applied to all chromosomes but the ten fittest ones (elitism) and the six clonal descendants that had already been mutated. The mutation rate was then high (30%) to prevent premature convergence and any mutation was generated by selecting a random value in the entire range of the model parameter. The algorithm was run until convergence was reached. Finally, a simple hill-climbing algorithm is applied to the best chromosome [50] to look for any minor improvement in the likelihood.

The high number of replications and sample sizes of human and vectors implied stability of the average estimates *P*_*H*_, *P*_*V*_ and *F*_*mix*_ after 1,000 replications (standard errors by bootstrap < 0.5%), which suggest consistency of the estimators [53]. Consequently, we applied standard likelihood methodologies to statistical analysis. The simulated likelihoods of the optimized models were compared using the Likelihood Ratio Test. In addition, a confidence interval for the *F* parameter was estimated using the profile likelihood method with profile likelihood approximated from GA searches where *F* was increased from 0.1 to 1 by 0.1 steps.

### Uncertainty and sensitivity analyses

An uncertainty analysis was performed in order to test how variation of different parameter values in their biological ranges affects the predictions of *F*_*mix*_. Particularly, the *F*_*mix*_ was obtained for each one of 1,000 uniformly distributed samples of the parameter space. We used a Latin hypercube sampling scheme as in [54]. The probability of predicting an *F*_*mix*_ as high as the observed (*F*_*mix*_^*Predicted*^ ≥ *F*_*mix*_^*Observed*^) was calculated.

A sensitivity analysis was performed to determine the impact of variation in the parameter values on the predictions of the best model. For each parameter, we used two standard measures of sensitivity; a coefficient of determination (R^2^) and a Pearson correlation coefficient (r) that were calculated between the parameter values and the estimated frequency of mixed-infection *F*_*mix*_. In addition, a variance-based sensitivity analysis to determine the total effect index (S_Ti_) which provides a measure of the contribution of each parameter to the variance of *F*_*mix*_. S_Ti_ was estimated by using the Jansen estimator and a Latin hypercube sampling of the parameter space with one-hundred matrices generated by radial sampling according to [55]. The values of R^2^, r, and S_Ti_ obtained for each parameter were normalized by dividing by the sum of values for all the parameters in order to be compared in a single graph.

### Robustness analyses

Since heterogeneity in exposure between hosts can potentially influence the rate of infection and reinfection in humans, we tested if the outcomes of our model selection approach were robust to the existence of between households heterogeneity in vector distribution. We introduced such heterogeneity in our full protection model to establish if those variations in vector abundance could lead to a better fit. Heterogeneity in vector abundance was modeled by using a negative binomial distribution with a mean number of vectors per house (*V*_*mean*_) and an aggregation parameter (*k*_*V*_). The range of *k*_*V*_ values was set to 0.01–1000. A value of *k*_*V*_ = 0.01 with *V*_*mean*_ = 100 implies that 50% of the houses are not infested, while the other 50% households receive all the 10000 vectors, with 12% of houses being infested by >500 vectors. On the other hand, a value of *k*_*V*_ = 1,000 with a *V*_*mean*_ = 100 implies little aggregation as about 99.5% of the houses will have at least 80 vectors.

Since dogs represent a well-known risk factor for *T*. *cruzi* transmission to humans [38], we analyzed if the inclusion of such a domestic reservoir could substantially change our estimates of the frequency of mixed infections. The ‘FP’ and the ‘APW + PF’ models were thus re-fitted to the data (using the hybrid GA method) while up to two reservoir individuals were added to each house. Vector preference for human was then varied between 0.3 and 0.7 according to observed levels of human blood index [40], and the rate of mortality of reservoirs was varied between 0.0009 and 0.0027 to account for an average lifespan of 1–3 years. The per-contact probability of transmission from vectors to reservoirs (*T*_*V→R*_) was varied between 2.6 x 10^−4^ and 2 x 10^−2^, and the per-contact rate of transmission from reservoirs to vectors (*T*_*R→V*_) was set to 0.49 according to available estimates [43]. In addition, we addressed the possibility of a heterogeneous distribution of reservoirs per house. In this regard, we implemented a negative binomial distribution with the mean number of reservoirs per house (*R*_*mean*_) and the aggregation parameter *k*_*R*_. The range of values for *k*_*R*_ was set to 1–10,000. *k*_*R*_ = 1 and *R*_*mean*_ = 1 implies that a mean of 50% of the houses will have no reservoirs.

### Estimation of the frequency of effective re-inoculations and reinfections

We distinguished effective re-inoculation, i.e. the entrance of the parasite in an infected human by avoiding the first barriers of defense (skin and innate immune response) from reinfection that follow re-inoculation. Such reinfection only occurs if the established adaptive immune response fails to clear the re-inoculated parasite. To infer on each of these stages we calculated both the frequency of re-inoculations and the frequency of reinfections.

The number of re-inoculations during a *t*-days period of time (*I*_*r*_) was assumed to follow a binomial distribution

$$I_{r}∼B(t\cdot n,\;T_{V}{}_{\to H})$$ (1)

where *n*, the number of potential infective contacts per day and per human, is given by

$$n=V\cdot B\cdot P_{V}/H$$ (2)

Accordingly, the mean number of effective re-inoculations (*Ī*_*r*_) was calculated as

$${\bar{I}}_{r}=t\cdot n\cdot T_{V}{}_{\to H}$$ (3)

We used Eq 3 to estimate the mean number of effective re-inoculations during the acute phase window (i.e. *t* = 60 days), and to predict the total number of effective re-inoculations by replacing *t* by the exposure time per year (180 days) multiplied by the number of years lived after infection.

Similarly, the average number of reinfections during the chronic phase (*Ī*_*reinf*_) was estimated from the expectation of a Binomial distribution where *t* stood for the duration of the chronic stage and *T*_*V→H*_ was lowered by the rate of protection failure rate (*F*), so that:

$${\bar{I}}_{reinf}=t\cdot n\cdot F\cdot T_{V}{}_{\to H}$$ (4)

## Results

### Full protection hypothesis is an unlikely explanation for the observed frequency of mixed infections

We first assessed the ‘Full Protection’ hypothesis using our ABM set with the *FP* transmission rule that does not allow for any reinfection (Fig 1B). The best fit of the *FP* model to the *P*_*H*_, *P*_*V*_ and *F*_*mix*_ observed in the rural villages (Table 2) is shown in Table 3 (see also S1 Fig and S1 Table for details about parameter estimates). The model predicted an *F*_*mix*_ substantially lower than the value observed in our study area (Table 3). Even when parameters values were allowed to vary within their biologically plausible ranges, the probability of predicting an *F*_*mix*_ ≥ *F*_*mix*_^*observed*^ remained very low (p = 0.002). Taken together, these analyses strongly suggest that the level of mixed infections measured in the villages of Chaco is unlikely to be reached if full protection is assumed and no reinfection is possible.

**Table 3:** Predicted prevalences, probabilities and Log-Likelihoods for different fitted models.

		Models							
	Observed	*FP*	*APW*	*PF*	*APW+PF*	*FP + k*_*V*_	*FP*_*+reservoir*_	*APW+PF* _*+reservoir*_	*FP*_*+ reservoir +*_ *k*_*R*_
***P*_*H*_**	0.441	0.42	0.43	0.43	0.43	0.46	0.45	0.44	0.42
***P*_*V*_**	0.17	0.21	0.18	0.18	0.17	0.21	0.19	0.17	0.23
***F*_*mix*_**	0.566	0.34	0.35	**0.55**	**0.57**	0.34	0.33	**0.54**	0.38
**Ln(L)**		-19.5	-17.1	-8.7	-8.6	-19.3	-18.8	-8.7	-18.5
**p(LR) ^1^**		-	0.03	3.3 x10^-6^	1.9 x10^-5^	*na*	*na*	*na*	*na*
**p(*F*_*mix*_*≥F*_*mix*_^*observed*^*)*^2^**		0.003	0.001	0.475	0.501	<0.001	<0.001	0.276	<0.001

*P*_*H*_, mean prevalence of infected humans. *P*_*V*_, mean prevalence of infected vectors. *F*_*mix*_, mean frequency of humans with mixed infections. *k*_*V*_, aggregation parameter for heterogeneous distribution of vectors per house. *K*_*R*,_ aggregation parameter for heterogeneous distribution of reservoir hosts per house.

^1^Likelihood ratio test against model *FP*.

^2^Probability for the fitted model to predict an *F*_*mix*_ equal or higher than the observed value.

### Reinfections are rare in the acute phase of the disease

We evaluated if the hypothesis of reinfections during the acute phase window may provide a better explanation to the observed level of mixed infections. The ABM set with the *APW* rule of transmission (Fig 1B) provided a slightly better fit than the model with no reinfection, as indicated by a marginally higher likelihood (Table 3). However, this second model did not allow for a major increase in the predicted value of *F*_*mix*_ that remained similar to the value predicted in the absence of reinfection. The uncertainty analysis confirmed that trend as the probability of predicting an *F*_*mix*_ ≥ *F*_*mix*_^*observed*^ remained very low (p = 0.004) when parameters values were varied within their biological range. Such a conclusion is better understood when quantifying the actual risk of reinfection during the acute phase, which can be made using Eq 1 and the estimated values of the model parameters (S1 Table). This risk of reinfection is very low as it only equals 0.0061, which implies one reinfection every 164.2 primary infections.

### Protection failure of the immune response explains the high frequency of mixed infections

**Fig 2:**
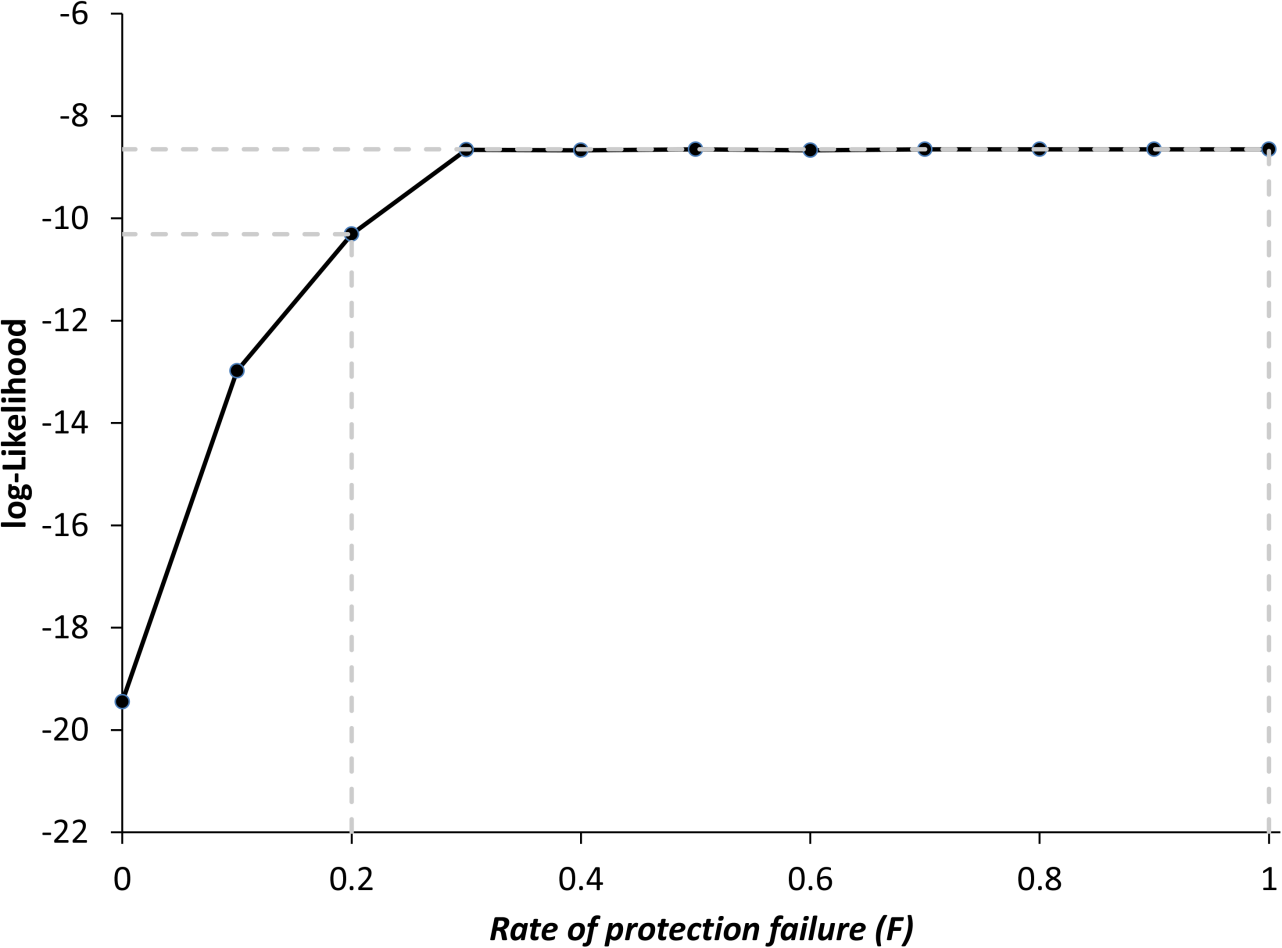
Approximated profile likelihood for the rate of protection failure (*F*) in the model *PF*. Dashed lines represent the lower and upper limit of the range of F values providing a good fit to the data.

We evaluated if the protection failure in the chronic stage (model *PF*, Fig 1B) can explain the high levels of mixed infection observed in our study area. The likelihood of the model *PF* was indeed significantly higher than the likelihood of the basic *FP* model (Table 3). In addition, the fitted model predicted an *F*_*mix*_ = 55% that nearly matched to 56% observed in the study area (Table 3 and S1 Fig). The best fit was obtained for a rate of protection failure (*F)* equal to 0.77, although Fig 2 indicates that a similar description of the data could be obtained for a large range of *F* values (*F* ≥ 0.2) as the Log-likelihood is not reduced by more than 2 (the threshold used to derivate confidence intervals of MLE estimates). As a matter of fact, when the value of *F* is increased, the estimates of the probabilities of transmission of a mixed infection (*T*_*mix*,*H->V*_ and *T*_*mix*,*V->H*_) take on larger values to match the observed frequency of mixed infection, although these variations remain bounded within 0.65 (when *F* = 1) and 0.90 (when *F* = 0.2). Accordingly, a safe outcome of the fitting of the *PF* model is that, the rate of protection failure must be larger than 20% to reproduce the frequency of mixed infection, i.e. at least 1 out of 5 re-inoculations typically lead to reinfection.

**Fig 3:**
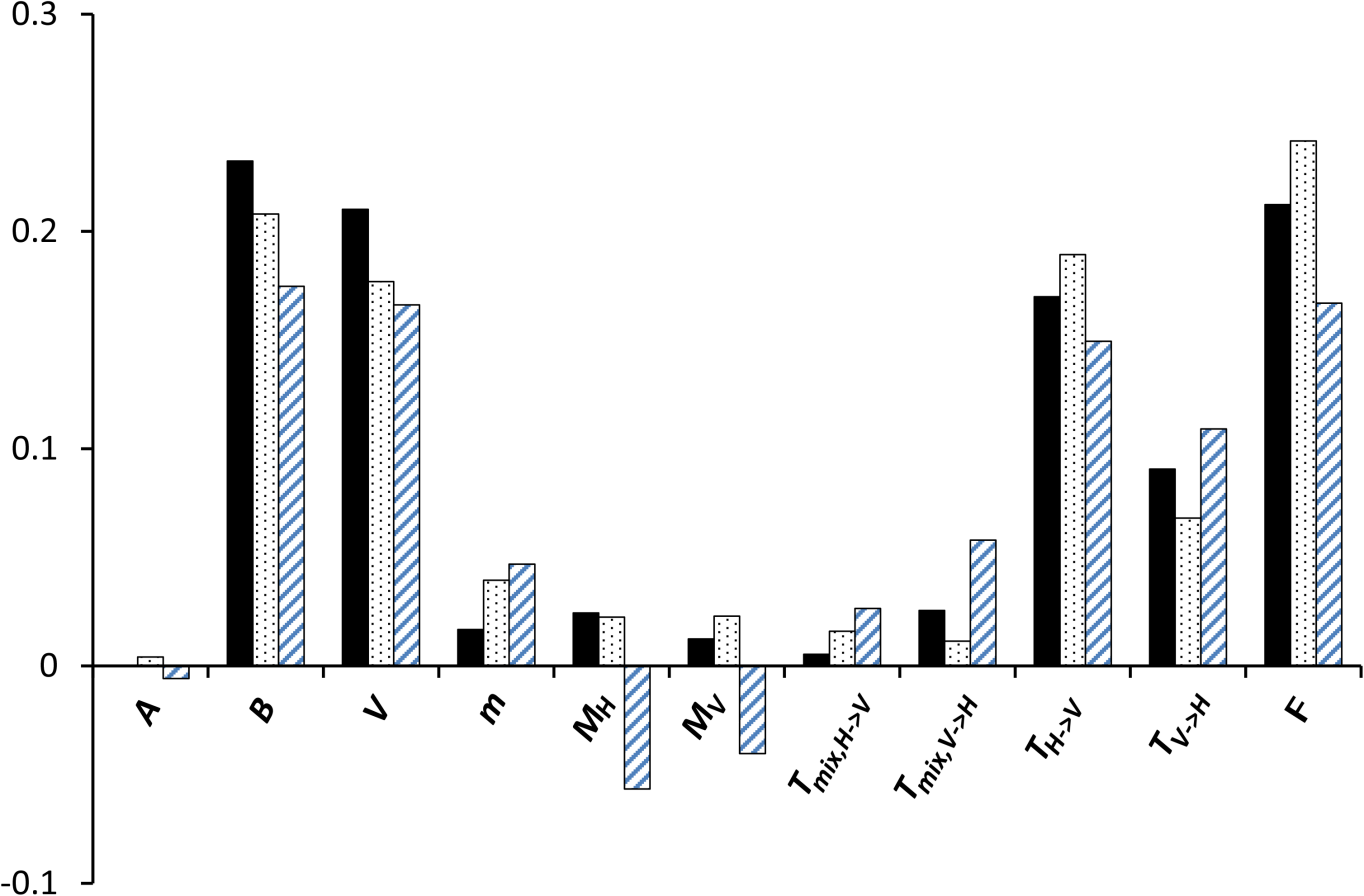
Sensitivity analysis of the frequency of mixed-infections (*F*_*mix*_) predicted by the best model (*APW + PF*). The sensitivity of the prediction to each parameter is quantified by three standard and normalized measures: R^2^ (black bars), Pearson correlation coefficient (dashed bars) and total effect index (S_Ti_) (dotted bars). The negative values of the coefficient of correlation indicate a decrease in the expected frequency of mixed-infection when the parameter value is increased.

The addition of the acute phase window (model *APW + PF*) did not provide a significantly better fit than the *PF* model (Table 3). This is consistent with the outcome of the analysis of the *APW* model where reinfections in the acute phase window were shown to be of negligible importance. It is also consistent with the best fit of model *APW + PF* that predicted an average of 98.4% (SE = 0.2%) of the reinfections occurring in the chronic phase. The *F*_*mix*_ predicted by the model was sensitive to variations in some parameter values. As expected, the prediction of the model was most sensitive to variations in the *F* parameter that has a direct effect in increasing *F*_*mix*_ (Fig 3). Other parameters with high effect on the *F*_*mix*_ included the number of vectors per house, the biting rate and transmission parameters (*T*_*V->H*_ and *T*_*H->V*_) whose variations were positively related to *F*_*mix*_. Despite such sensitivities, the uncertainty analysis showed that the model predicted high levels of reinfections in a large part of the parameter space (p = 0.73 for *F*_*mix*_ ≥ *F*_*mix*_^*observed*^ in the uncertainty analysis). This confirmed the model with protection failure does provide much better predictions than any of the considered alternatives.

### Repeated re-inoculations and reinfections are rare events

**Fig 4:**
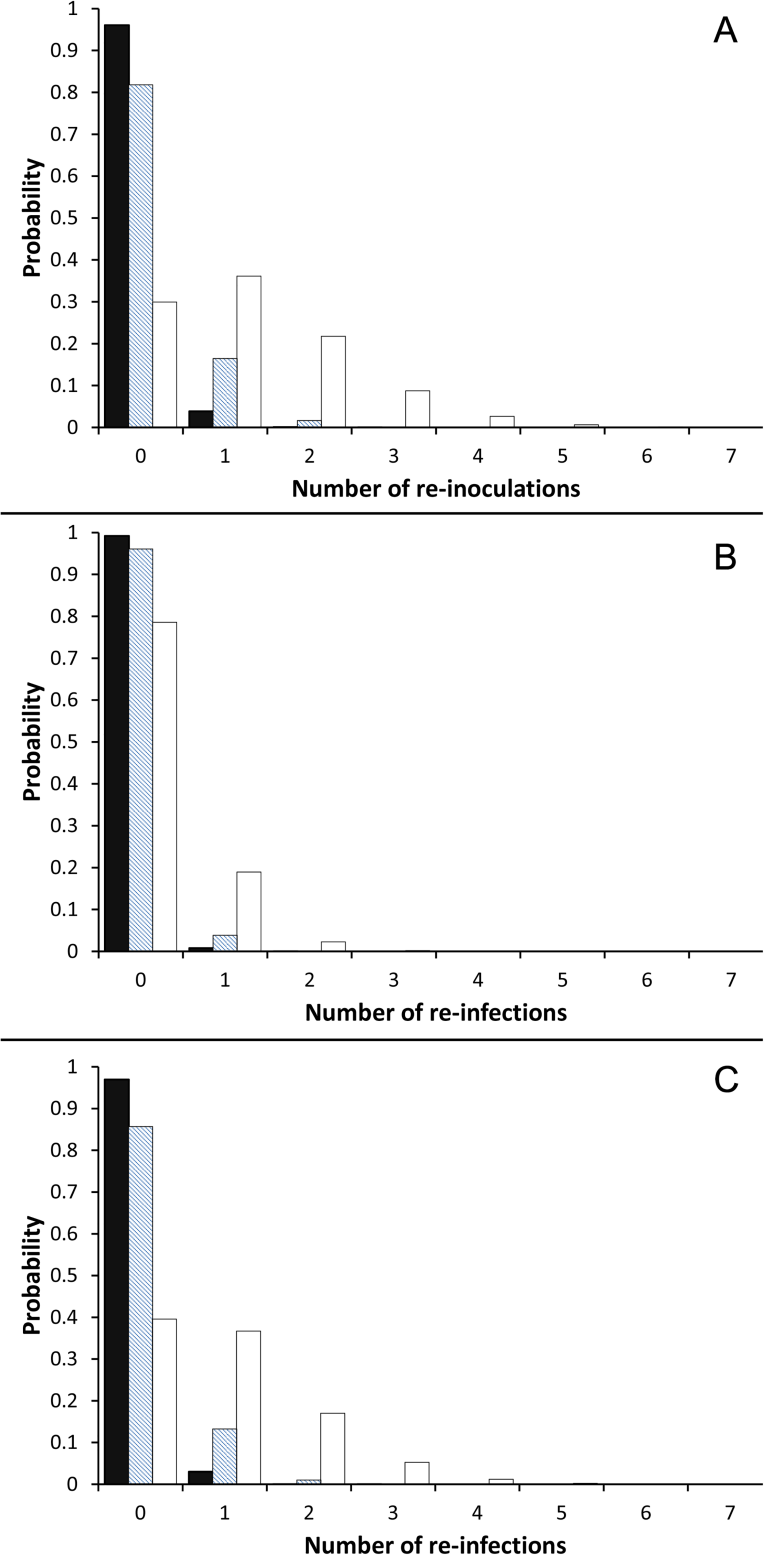
Frequency of re-inoculations and reinfections based on the best model (*PF)*. (A) Distribution of the number of effective re-inoculations per individual one-year (black bars), five-years (dashed bars) and thirty-years (white bars) after the primary infection. (B and C) Distribution of probability of reinfection one-year (black bars), five-years (dashed bars) and thirty-years (white bars) after the primary infection considering (B) *F* = 0.2 and (C) *F* = 0.77.

We took advantage of the predictions of the model *PF* to derive the expected frequency of effective re-inoculations and reinfections. Interestingly, 4%, 18% and 70% of individuals were found to be re-inoculated with the parasite at least once after 1, 5 and 30 years following the primary infection (Fig 4A). Since the corresponding risk of reinfection clearly depends on the rate of failure of the immune response, we derived the distributions of the number of reinfections for both the best estimate of *F* (*F* = 0.77) as well as for the lower (*F* = 0.2) and upper (*F* = 1) limit of the range of *F* values that provided a good fit to the data (Fig 3). The risks of individual reinfection after 1, 5 and 30 years of chronic infection were shown to be 3%, 14%, 60% when calculated with the best estimate of *F* (Fig 4C). They decreased to 0.8%, 4% and 21% when estimated from lowest allowed rate of failure (Fig 4B). On the contrary, when the immune response was considered to be totally inefficient (*F* = 1), so that re-inoculations are strictly equivalent to reinfections, these risks increased to their maximum and reach 5%, 18% and 70% (Fig 4A). In all cases, the number of per individual reinfections hardly ever exceeded 4 (Fig 4A–4C). Accordingly, despite a rate of protection failure estimated to be at least 20%, reinfections remain rare events in the rural villages of our study area.

### Robustness analysis

Our models assume non-heterogeneity in exposure to infection and re-infection e.g. each house has the same number of vectors. In order to evaluate if heterogeneity in exposure may bias our conclusions, we have modified the *FP* model by including heterogeneity in the number of vectors per house (*FP* + *k*_*V*_). Such heterogeneity was modeled by using a negative binomial distribution. The best *FP* + *k*_*V*_ model did not fit the data better than the *FP* model (Table 3). Consequently, heterogeneity in the number of vectors per house cannot explain the high frequency of mixed infections.

To evaluate if the inclusion of animal reservoirs may bias our conclusions by causing high frequency of mixed infections in vectors and subsequently in humans, we added up to two reservoir individuals per household to the basic model (*FP)* and to our best model (*PF*) that were then fitted again. These more complex models did well predicting a low prevalence of infection in reservoirs, which was concordant with observed values in the study area, but they did not provide a significantly better fit to the frequency of mixed-infection in humans than the previous version of models *FP and PF* (Table 3). In addition, including heterogeneity in the number of reservoirs per house using a negative binomial distribution does not improve the fit of the *FP* model (Table 3).”

## Discussion

Despite the large-scale international control initiatives launched during the nineties to eliminate triatomine populations, human infection with *T*. *cruzi* remains predominantly associated with vector transmission [56]. In areas where human communities are highly exposed to vectors, the annual number of potentially infectious bites per human can potentially reach 13–340 [39]. With such levels of risk, such as in the Chaco region, not only the prevalence of Chagas disease can remain as high as 55–97% (e.g. [57], [58]), but we also expect individuals will face multiple reinfections as the immune response mounted during primary infection is likely not to be fully protective [59]. While such reinfections are thought to have important effects on cardiomyopathy [28, 29, 32] and the probability of congenital transmission [60], little is known about their frequency in rural communities. To determine the amount of reinfections per individual thus remains a key challenge to improve our understanding of the disease dynamics and its impact on the populations. For obvious ethical reasons, reinfections in humans cannot be experimentally evaluated, and estimates of the rate and timing of *T*. *cruzi* reinfections thus rely on indirect approaches based on a combination of epidemiological data and models of *T*. *cruzi* transmission. Although various models have been developed to explain the prevalence of Chagas disease in humans [38–40, 46, 47, 61–65], most of them typically consider a single parasite and provide no prediction about the frequency of reinfection (see [47] for a review). In this paper, we developed an agent-based modelling approach that account for infection by multiple *T*. *cruzi* DTU, and therefore allows evaluating the frequency and timing of reinfections in humans.

Using epidemiological field data from two rural villages from Chaco (Argentina) we identified two models of transmission (*PF* and *APW* + *PF*) that reproduce the epidemiological patterns observed in the field, for parameter values within the range of previous estimations [39,40,43,66–69]. This accuracy and the sensitivity analyses we performed suggest that while the models remained parsimonious, they included the most relevant factors underlying the dynamic of transmission in the studied area. The results derived from these models allowed us to infer about the rate and timing of *T*. *cruzi* reinfections from epidemiological field data.

The first important outcome of our study is to show that only mixed primary infections cannot explain the observed frequency of mixed infections in humans. Not only reinfections in the chronic phase are necessary to explain the epidemiological data in the two studied localities of the Chaco area, but also, for such reinfections to be frequent enough, the rate of failure of the protective immunity mounted after the primary infection by *T*. *cruzi* has to be at least 20%. Such estimate clearly shows that in humans, just as it has been shown experimentally in mice [59] and dogs [23], reinfections are far from being fully prevented by adaptive immunity. We shall point out that our estimate of a rate of failure of the adaptive immune response greater than 20% was obtained under the simplifying assumptions that all DTUs confer the same protection against reinfections and that different lineages have the same probabilities of transmission. Considering heterogeneity in host immunity and/or filtering of the different DTUs [70–73] would typically reduce the predicted frequency of mixed infections, and a higher protection failure rate will then be required to fit the frequency of mixed infections observed in the study area. On the other hand, the high rate of mixed infections in people from El Palmar and Las Leonas was detected using highly sensitive hybridization assays with specific probes from minicircle hypervariable regions (mHVRs) [37]. This marker, which allows DTU assignment directly from biological samples, has revealed high frequency of mixed infections in other epidemiological contexts [74–78]. A possible drawback of such a marker could be a reduced specificity. However, even if the method was not fully specific and over-estimated mixed infections, accounting for a typical 80% of specificity would lead to a true frequency of mixed infections reaching 46% (instead of 56.6%, see Table 2). Such a frequency would thus remain high and, again, only models taking into account a protection failure during the chronic stage (i.e. *PF* and *APW + PF*) would be able to predict such level of mixed infections (see Table 3). Our predictions about the failure of the adaptive immune response and the rate at which it allows reinfection thus seems robust outcomes of this study, and those quantitative findings are consistent with experimental measurements of the rate of (non-) immunization after priming of the immune response that were shown to reach 40–50% in mice [79], 47%-61% in Guinea pigs [80] and 46% in dogs [81].

The second main outcome of the model is to provide indirect estimates of the frequency distribution of reinfection in humans. It has been repeatedly proposed that infected people living in endemic areas suffer from frequent reinfections, and that such reinfections increase the strength of cardiomyopathy in humans [28, 29, 32], just as they do in mice [20–22, 30, 31]. Although those epidemiological studies were based on sound statistical comparisons between individuals with different levels of exposure to vectors, none of them provided estimates of the actual level of vector-human contacts and its associated number of reinfection events per person. Fitting our model of *T*. *cruzi* transmission to eco-epidemiological data collected in two villages of Chaco, we provided the first estimate of the frequency of human reinfection. We indeed showed that 3% (0.8%-5%), 14% (4%-18%) and 60% (21%-70%) of individuals are re-infected after 1, 5 and 30 years of the chronic stage of the disease. Those quantitative figures are partially explained by the very low probability of stercorarian transmission of *T*. *cruzi*. Our estimation of such probability of transmission (4.9 x 10^−4^ for the *PF* model) is highly consistent with the rare estimates present in the literature [39, 40]. It seems indeed not fully appreciated that given the extremely inefficient mode of *T*. *cruzi* transmission, one cannot expect reinfections to happen even on an annual basis. Considering the 20–30% of infected people that develop a potentially life-threatening heart disease [56], and assuming that they correspond to the individuals suffering with the most reinfections, they would typically be re-infected 1–2 times every 10 years according to the estimates of this study. Such a frequency appears unlikely to be enough to sustain antigen exposure, the consequent inflammatory response at a high chronic level, and a resulting higher cardiac morbidity. Surely, we must point out that our estimates were derived in a specific entomological context that can be better characterized by calculating the annual number of potentially infectious contacts per human, which we found to be equal to 86 in our study area. This value lies slightly below average for *T*. *infestans* and it represents a fourth of the maximal value reported in the literature [39]. We thus simulated a four-fold increase in this standard measure of exposure to vectors. Noteworthy, such an exceptional level of exposure made the 20–30% most re-infected individuals to be re-infected 2–5 times every 10 years, which still not correspond to yearly reinfections.

A last outcome of our study came as a by-product of our analysis of the robustness of our modeling outcome to the presence of reservoirs. Dogs have been demonstrated to be a major risk factor of house infestation not only for key vector species adapted to human households, such as *T*. *infestans* [38, 82, 83], but also for non-domiciliated species such as *T*. *dimidiata* [84]. They have further been shown to be intra-domiciliary reservoirs whose presence increases the prevalence of infection in *T*. *infestans* [38, 85]. By contrast, our modeling showed that, in the eco-epidemiological context encountered in El Palmar and Las Leonas, the presence of 1–2 reservoirs only slightly increases the prevalence of *T*. *cruzi* infection in vector and human, and they were not necessary to explain the observed levels of mixed infections. These constitute indirect evidence that dogs may not be significant intra-domiciliary reservoirs in these villages, which is consistent with their prevalence of infection with *T*. *cruzi* being twice as low as in humans (Table 1), and with the inhabitants reporting that dogs do not sleep indoors. The situation in the two studied villages thus contrasts with reported studies on several other rural villages from northwestern Argentina, where >40% of dogs were infected and considered to be relevant for the household transmission cycle [38, 85]. This suggests that the variation in the actual impact of potential reservoirs can be large enough for keeping dogs outside of dormitories to be efficient in lowering transmission to human, a control strategies that was earlier suggested by a different modeling approach [38].

As a concluding remark, we would like to emphasize that, although the immune system can prevent high parasitaemia after *T*. *cruzi* re-inoculation (as most experimental vaccines against *T*. *cruzi* do), this does not appear to be efficient enough to prevent reinfections. The latter would, nonetheless, remain rare events as *T*. *cruzi* stercorarian transmission put a providentially strong limit on human primary and secondary infections.

## Supporting information

S1 Fig *P*_*H*_, *P*_*V*_, *F*_*mix*_ predicted by different fitted models. (PDF)

S1 Table Estimated parameters for different fitted models. (DOCX)
