# Supplementary material for: Epidemiological modeling of Trypanosoma cruzi: Low stercorarian transmission and failure of host adaptive immunity explain the frequency of mixed infections in humans
Source: PLoS Comput Biol. 2017 May 8;13(5):e1005532. doi: 10.1371/journal.pcbi.1005532 (PMC5440054; doi:10.1371/journal.pcbi.1005532)
Supplement: S1 Fig — (PDF) [file pcbi.1005532.s001.pdf]

Model *FP* (full protection)

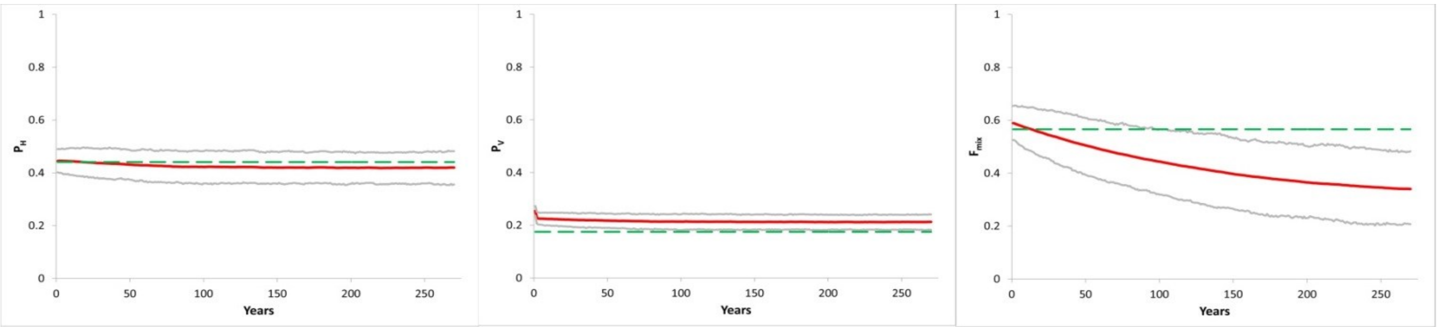

Model APW (acute phase window)

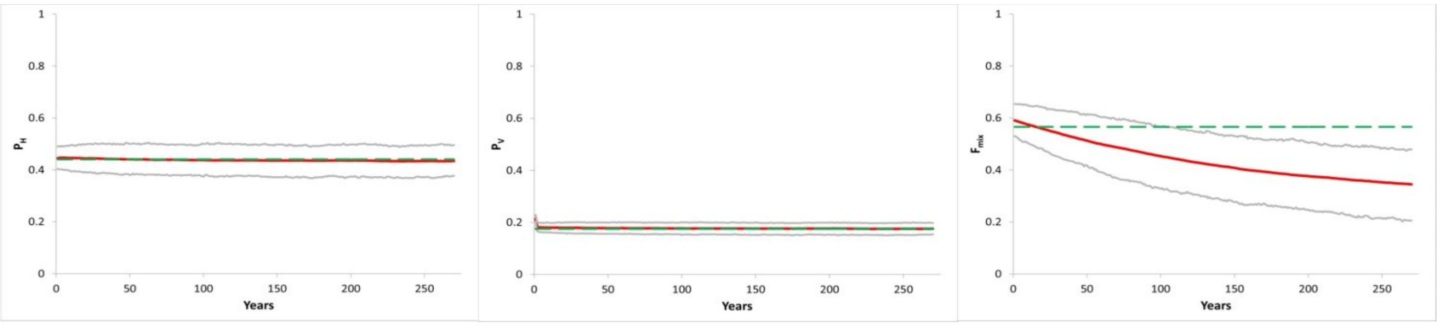

Model *PF* (protection failure)

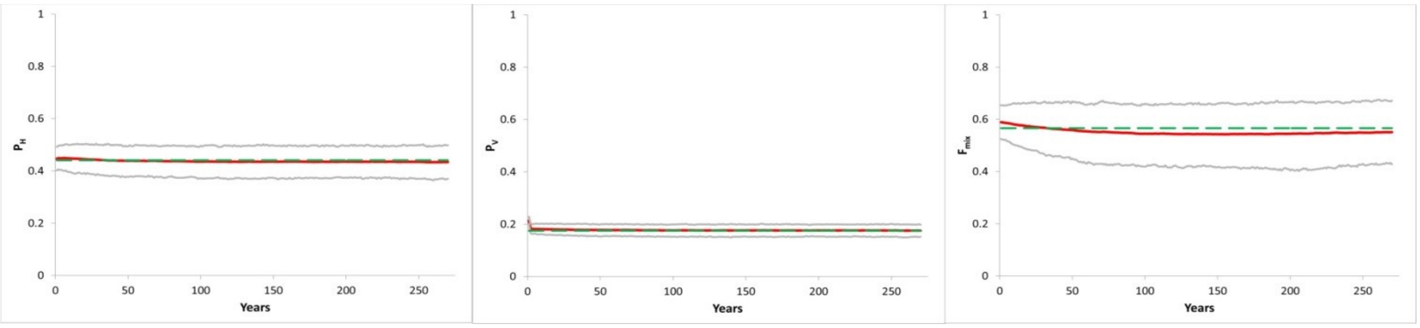

Model *APW + PF* (Acute phase window + protection failure)

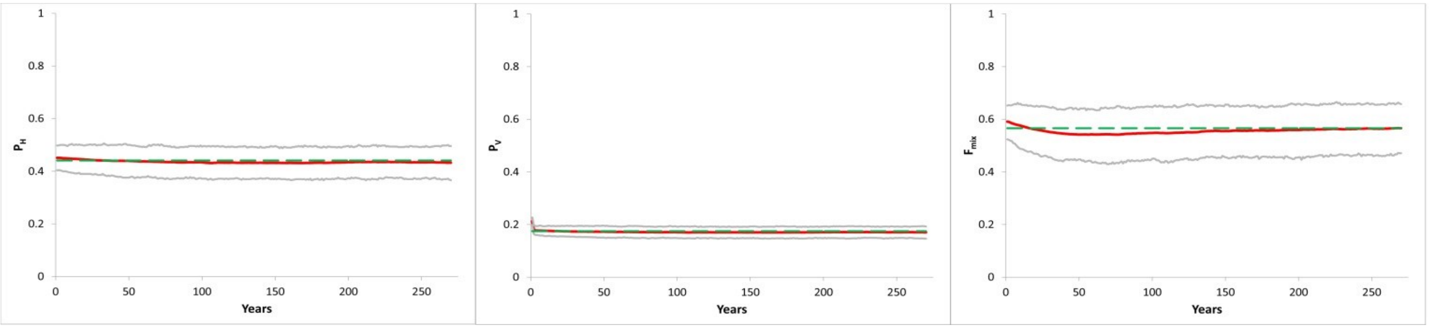

Red line= Average of 1000 runs with different seeds

Gray lines = lower, 2.5 percentile. Upper, 97.5 percentile

Dotted Green line = value observed in the rural villages
