# Supplementary material for: Epidemiological modeling of Trypanosoma cruzi: Low stercorarian transmission and failure of host adaptive immunity explain the frequency of mixed infections in humans
Source: PLoS Comput Biol. 2017 May 8;13(5):e1005532. doi: 10.1371/journal.pcbi.1005532 (PMC5440054; doi:10.1371/journal.pcbi.1005532)
Supplement: S1 Table — (DOCX) [file pcbi.1005532.s002.docx]

Supplementary Table 1. Estimated parameter values for the fitted models, with the best model appearing in the shaded column.

|  | **Models** | | | | | | | | |
| --- | --- | --- | --- | --- | --- | --- | --- | --- | --- |
| **Parameters** | FP | APW | PF | PF + APW | FP + *k_V_* | FP *+Reservoir* | FP +*Reservoir* + *k_R_* | PF + APW +*Reservoir* |  |
| Protection failure rate in the chronic phase (*F)* | 0 (fixed) | 0 (fixed) | 0.769 | 0.68 | 0 (fixed) | 0 (fixed) | 0 (fixed) | 0.937 |  |
| Acute phase duration (*A)* | 0 (fixed) | 48 | 0 (fixed) | 52 | 0 (fixed) | 0 (fixed) | 0 (fixed) | 10 |  |
| Feeding rate of vectors on mammals per day *(B)* | 0.157 | 0.09 | 0.19986 | 0.103 | 0.231 | 0.092 | 0.112 | 0.149 |  |
| Mean number of vectors per house *(V)* | 55 | 84 | 67 | 113 | 50 | 222 | 71 | 284 |  |
| Probability of transmission from H to V (*T_H->V_)* | 0.0433 | 0.048 | 0.026 | 0.042 | 0.025 | 0.049 | 0.011 | 0.026 |  |
| Probability of transmission from V to H (*T_V->H_)* | 0.00042 | 0.00051 | 0.00049 | 0.00063 | 0.00045 | 0.00035 | 0.00041 | 0.00039 |  |
| Migration rate of vectors *(m)* | 0.018 | 0.0184 | 0.0197 | 0.017 | 0.017 | 0.019 | 0.018 | 0.016 |  |
| Mortality (and emigration) of humans *(M_H_*) | 8.23E-05 | 7.15E-05 | 0.000103 | 0.00013 | 9.85E-05 | 7.41E-05 | 7.12E-05 | 0.000126 |  |
| Mortality of vectors *(M_V_*) | 0.00628 | 0.00492 | 0.00708 | 0.00516 | 0.0059 | 0.00651 | 0.0046 | 0.00757 |  |
| Probability of transmission of a mixed infection from H to V (*T_mix,H->V_*) | 0.894 | 0.9 | 0.88 | 0.865 | 0.9 | 0.896 | 0.884 | 0.595 |  |
| Probability of transmission of a mixed infection from V to H (*T_mixV->H_*) | 0.9 | 0.9 | 0.644 | 0.702106 | 0.9 | 0.9 | 0.9 | 0.673 |  |
| Aggregation Parameter for Vectors (*k_V_*) | - | - | - | - | 292.4 | - | - | - |  |
| Mean number of Reservoirs per house *(R)* | 0 (fixed) | 0 (fixed) | 0 (fixed) | 0 (fixed) | 0(fixed) | 2 | 0.987 | 1 |  |
| Probability of transmission from R to V (*T_R->V_*) | - | - | - | - | - | 0.49 | 0.22 | 0.036 |  |
| Probability of transmission from V to R (*T_V->R_*) | - | - | - | - | - | 0.00052 | 0.009 | 0.0026 |  |
| Mortality of reservoirs *(M_R_*) | - | - | - | - | - | 0.0015 | 0.0018 | 0.0018 |  |
| Human blood index *(BI)* | - | - | - | - | - | 0.536 | 0.648 | 0.414 |  |
